# MicroRNA-5110 regulates pigmentation by cotargeting melanophilin and WNT family member 1

**DOI:** 10.1096/fj.201800040R

**Published:** 2018-05-07

**Authors:** Shanshan Yang, Bo Liu, Kaiyuan Ji, Ruiwen Fan, Changsheng Dong

**Affiliations:** College of Animal Science and Veterinary Medicine, Shanxi Agricultural University, Taigu, China

**Keywords:** MLPH, WNT1, RAB27A, MYO5A

## Abstract

Mammalian pigmentation requires the production of melanin by melanocytes and its transfer to neighboring keratinocytes. These complex processes are regulated by several molecular pathways. Melanophilin (*MLPH*) and WNT family member 1 (*WNT1*), known to be involved in melanin transfer and melanin production, respectively, were predicted to be targets of microRNA-5110 using bioinformatics. In the current study, we investigated the effects of microRNA-5110 on pigmentation in alpaca (*Vicugna pacos*) melanocytes. *In situ* hybridization identified high levels of microRNA-5110 in the cytoplasm of alpaca melanocytes. Luciferase activity assays confirmed that *MLPH* and *WNT1* were targeted by microRNA-5110 in these cells. Overexpression and knockdown of microRNA-5110 in alpaca melanocytes downregulated and upregulated MLPH and WNT1 expression at the mRNA and protein levels, respectively. In addition, overexpression and knockdown of microRNA-5110 in alpaca melanocytes decreased and increased, respectively, the mRNA levels of the melanin transfer-related genes, rat sarcoma (RAS)–associated binding (*RAB27a*) and myosin 5a (*MYO5a*); the mRNA levels of microphthalmia-associated transcription factor (*MITF*), tyrosinase (*TYR*), and tyrosinase-related protein (*TYRP*)*1*; and the production of total alkali melanin and pheomelanin. In contrast, overexpression and knockdown of microRNA-5110 increased and decreased the mRNA levels of *TYRP2*, respectively. Overexpression of microRNA-5110 also increased eumelanin. These results indicate that microRNA-5110 regulates pigmentation in alpaca melanocytes by directly targeting *MLPH* and *WNT1* to affect eumelanin production and transfer.—Yang, S., Liu, B., Ji, K., Fan, R., Dong, C. MicroRNA-5110 regulates pigmentation by cotargeting melanophilin and WNT family member 1.

Alpaca (*Vicugna pacos*) is a fiber-producing camelid species with 22 natural hair colors, which are determined by a combination of genetic and endocrinological factors (1). An increasing number of genes have been reported to influence hair color in mammalian species. Among them, microphthalmia-associated transcription factor (MITF) (2) is an important transcription factor that regulates the key limiting enzymes of melanogenesis, tyrosinase (TYR) and tyrosinase-related protein (TYRP)1 and 2.

MicroRNAs are evolutionarily conserved noncoding 22–25 nt RNA molecules that control the expression of their target genes at the post-transcriptional level (3). Studies on the functions of microRNAs in melanogenesis are limited, although many microRNAs have been shown to play significant roles in melanoma (4, 5). Deep sequencing comparisons of alpaca skin with white *vs.* brown coat color identified several potential microRNAs, which could have novel functions in melanogenesis (6). MiRBase and Targetscan predicted that melanophilin (*MLPH*) and WNT family member 1 (*WNT1*) are 2 target genes of microRNA-5110. To explore the influence of microRNA-5110 on alpaca pigmentation, the functional roles of microRNA-5110 in alpaca melanocytes were investigated and presented in this study.

## MATERIALS AND METHODS

Housing and care of alpacas and collection of skin samples were approved by the Animal Experimentation Ethics Committee of Shanxi Agricultural University [2017(050)].

### *In situ* hybridization

The alpaca melanocytes used in this study were established in our lab and were maintained as previously described (7). For *in situ* hybridization, the cells were fixed in 4% paraformaldehyde in 0.1 M (PBS; pH 7.2–7.6) with 1:1000 diethylpyrocarbonate for 30 min, and the slides were washed 3 times with 0.1 M PBS (pH 7.4) for 2 min/wash. The cells were treated with 40 g/ml proteinase K (Roche Applied Science, Penzberg, Germany) and fixed in 1% paraformaldehyde for 10 min. Digoxigenin-labeled probes (3 pM) were diluted in 20 μl hybridization buffer, applied to the slides, and allowed to hybridize at 37°C overnight. The slides were washed at 37°C in 2× SSC solution and incubated with alkaline phosphatase–conjugated mouse anti-digoxigenin antibody (1:1000; Roche Applied Science) for 2 h at 37°C. Alkaline phosphatase was detected with 3,3′-diaminobenzidine as a substrate. The microRNA-5110 probe sequence was 5′-AATTCCACCACCCTCTACCTCCTCC-3′, and the scrambled sequence was 5′-GTGTAACACGTCTATACGCCCA-3′ (Bio-High Technology, Shijiazhuang, China).

### Construction of plasmids

The microRNA-5110 expression and inhibitor plasmids were constructed by inserting an oligonucleotide corresponding to the sequence of pre-microRNA-5110 into the mammalian expression vector, pcDNA6.2-GW/EmGFPmiR (Thermo Fisher Scientific, Waltham, MA, USA), resulting in the CMV promoter driving the expression of green fluorescent protein and microRNA-5110. A negative control (NC) plasmid was also constructed with a scrambled pre-microRNA-5110 sequence. The luciferase reporter plasmids were constructed by cloning the 3′-UTR sequences of alpaca *MLPH* or *WNT1* into the dual luciferase pmirGLO vector (Promega, Madison, WI, USA). Partial sequences of alpaca *MLPH* and *WNT1* containing the microRNA-5110 binding sites were obtained by PCR of alpaca skin cDNA, by using primers containing *Sac*I and *Xho*I sites. The PCR products and vector were digested with *Sac*I and *Xho*I and ligated together to obtain the pmirGLO-MLPH-wild-type (WT) and pmirGLO-Wnt1-WT constructs. The microRNA-5110 binding sites in these plasmids were then mutated with a Site-Directed Gene Mutagenesis Kit (Beyotime, Shanghai, China), according to the manufacturer’s instructions, to obtain the pmirGLO-MLPH-mut and pmirGLO-Wnt1-mut plasmids. All constructs were confirmed by sequencing.

### Cell culture and transfection

Melanocytes were transfected with the pcDNA6.2-microRNA-5110 inhibitor or scrambled control plasmid using Lipofectamine 2000 (Thermo Fisher Scientific) according to the manufacturer’s instructions. Three days after transfection, the melanocytes were collected. Cell lysates and total RNA were prepared and subjected to Western blot and real-time quantitative PCR (qPCR) analyses, respectively.

### Dual luciferase assay for microRNA target validation

HEK293T cells were cultured in DMEM (Thermo Fisher Scientific) supplemented with fetal bovine serum (10%). For the luciferase reporter assay, 2 μg of the plasmids was cotransfected into HEK293T cells with Lipofectamine 2000. Luciferase activities in the transfected cells were measured with a Dual-Luciferase Reporter Assay Kit (Promega) 2 d after cotransfection. Firefly luciferase activity was normalized to *Renilla* luciferase activity, to adjust for transfection efficiency. Data are expressed as the mean relative luciferase activities ± sd (*n* = 3).

### Real-time qPCR for microRNA and mRNA

Total RNA was extracted from melanocytes with Trizol reagent (Thermo Fisher Scientific) according to the manufacturer’s instructions, and treated with DNase I (MilliporeSigma, Burlington, MA, USA). For mRNA quantification, 1 μg total RNA was converted to cDNA with a cDNA Synthesis Kit (Takara, Dalian, China) according to the manufacturer’s instructions. For microRNA quantification, cDNA was generated with a cDNA Synthesis Kit (Takara) with specific stem-loop primers and a common reverse primer, in accordance with a previously established method for real-time quantification of microRNA (8). For both analyses, qPCR was performed with Sybr Green PCR Master Mix (Takara) on a 7500 Fast Real-Time PCR System (Thermo Fisher Scientific). The primer sequences are listed in **Table 1**. Melting curves for each sample were analyzed to validate amplification specificity. All samples were run in triplicate, and the relative amounts of microRNA or mRNA were normalized to the amount of U6 or β-actin mRNA, respectively. Quantification of microRNA and mRNA transcript abundance was performed by using the comparative threshold cycle method (9). The significance of differences in the levels of microRNA and mRNA was determined by ANOVA (SPSS 11.5 Software; IBM, Armonk, NY, USA).

**TABLE 1:** MicroRNAs and mRNAs primer sequences

	Primer sequence, 5′–3′		
Gene	Forward	Reverse	Application
*MLPH*-WT	CGAGCTCTGTCACCTGTGCCCTGAACTGCTTG	GCCTCGAGGGTGGGTATGGTAAGGAACTCT	Luciferase reporter-WT
*MLPH*-mut	AAGCCCCATTGGTCCCAGGAAAGCAGGACC	TTCGGGGTAACCAGGGTCCTTTCGTCCTGG	Luciferase reporter-mut
*WNT1*-WT	GCGAGCTCTGTTGCGGTTCCTGATGT	GCCTCGAGCCTATGAGAAGCTGGGTAAA	Luciferase reporter-WT
*WNT1*-mut	ATGCCTCCCTCAGCCTGGACCCACCCCTTCCTG	TACGGAGGGAGTCGGACCTGGGTGGGGAAGGAC	Luciferase reporter-mut
MicroRNA-5110	ACACTCCAGCTGGGGGAGGAGGTAGAGGGTGGT	TGGTGTCGTGGAGTCG	Real-time PCR
Common-R	CGAGCAGTGCAGGGTCCGAGGT		RT-PCR
*U6*	CTCGCTTCGGCAGCACA	GTCGTATCCAGTGCAGGGTCCGAGGTATTCGCACTGGATACGACTCATCT	Real-time PCR
*MLPH*	ATACTTCTTCCCCGGCTTGT	CTGTTGGGGTTCCTCTGTGT	Real-time PCR
*WNT1*	ACCTGCTGACCGATTCCAAG	ACAGCCTCGGTTGACGATTT	Real-time PCR
*Rab27a*	TCACGACAGTCGGCATTG	GCTCTGGCTTCTTCCTCT	Real-time PCR
*Myo5a*	GGACTAAGAAAGCGAACC	TGAGCAGCCTGAATGAGA	Real time PCR
*MITF*	TCCCAAGTCAAATGATCCAG	GAGCCTGCATTTCAAGTTCC	Real-time PCR
*TYR*	GCTTTAGCAACTTCATGGGA	CTTGTTCTTCTCTGGGACAC	Real time PCR
*TYRP1*	GCCTTCTTTCTCCCTTC	CAGACCACTCGCCATT	Real-time PCR
*TYRP2*	AGCAGACGGAACACTGGACT	GCATCTGTGGAAGGGTTGTT	Real-time PCR
β-Actin	CTAAGGAGAAGGGCCAGTCC	CTCAAGTTGGGGGACAAAAA	Real-time PCR

WT, wild-type; mut, mutation.

### Western blot analysis

Protein samples were separated by 10 or 8% SDS-PAGE and transferred to PVDF membranes. The membranes were blocked with 5% skim milk for 2 h and incubated overnight at 4°C with the following diluted primary antibodies raised against MLPH (1:500), WNT1 (1:500), rat sarcoma (RAS)–associated binding 27a (RAB27a) (1:800), MYO5a (1:1000), MITF (1:800), TYR (1:1000), TYRP1 (1:1000), TYRP2 (1:1000), and β-actin (1:1000). After the membranes were washed 4 times with TBST for 5 min/wash, they were incubated for 1 h at 37°C with horseradish peroxidase–conjugated secondary antibodies raised against rabbit or mouse IgG (1:10,000). After the membranes were again washed with TBST 4 times for 5 min/wash, the bound antibodies were visualized using enhanced chemiluminescence. Immunoblots were scanned on a ChemiDOC XRS+ Imager (Bio-Rad, Hercules, CA, USA), and protein levels were quantified with Image-Pro Plus Software (Olympus, Tokyo, Japan).

### Immunocytochemistry

Melanocytes were washed 3 times (3 min/wash) with PBS, fixed in 4% paraformaldehyde, and incubated at room temperature in 3% hydrogen peroxide for 15 min to block the action of endogenous peroxidases. After three 5-min washes with PBS, the cells were immersed in bovine serum albumin at 37°C for 25 min and incubated at 4°C overnight in rabbit anti-MLPH antibody or rabbit anti-WNT1 antibody solution. After three 5-min washes with PBS, the cells were incubated with horseradish peroxidase–conjugated anti-rabbit IgG for 30 min at 37°C and observed under a microscope (Leica Microsystems, Buffalo Grove, IL, USA). For the NC, IgG was substituted for the primary antibody.

### Immunohistochemistry

Paraffin-embedded sections were dehydrated with increasing concentrations of ethanol (80–100%), followed by incubation in 3% hydrogen peroxide for 15 min at room temperature to block endogenous peroxidases. After the sections were washed with 0.1 M PBS 3 times for 15 min/wash, they were blocked for 20 min in PBS containing 5% bovine serum albumin at 37°C. The sections were then incubated at 4°C overnight with rabbit anti-MLPH or rabbit anti-WNT1 primary antibody (1:100 dilution). After 3 washes with PBS, the sections were incubated in horseradish peroxidase–conjugated goat anti-rabbit IgG (1:500 dilution) for 30 min at 37°C. After another washing with PBS 3 times, the sections were developed with 3,3′-diaminobenzidine, and the positive signals were observed with a Leica microscope (Leica Microsystems). For the NC, IgG was substituted for the primary antibody.

### Melanin measurement

Melanocytes were harvested and rinsed with PBS. For the assay of total alkali melanin, 1 ml of 0.2 M NaOH was added, and the absorbance of the solution was measured spectrophotometrically at 475 nm. For the assay of eumelanin, the cells were hydrolyzed in hot 30% hypophosphoric acid and hydriodic acid. After cooling, 50% ethanol was added, and the cell samples were centrifuged at 2234 *g* in a Model 4225 centrifuge (Associated Clinical Laboratories, Chicago, IL, USA; no longer manufactured) for 10 min. Insoluble eumelanic pigments were selectively solubilized in hot sodium hydroxide and hydrogen peroxide and the solution was cleared by centrifugation at 10,700 *g* for 1 min in a Sorvall Ultracentrifuge (Thermo Fisher Scientific). The absorbance of the supernatant was measured at 350 nm. For the assay of pheomelanin, the cells were solubilized in phosphate buffer (pH 10.5) and cleared by centrifugation at 10,700 *g* for 10 min before determination of the absorbance at 400 nm. The melanin levels were normalized to the total number of cells. All experiments were performed in triplicate.

### Statistical analysis

Data related to the levels of target microRNA, mRNA, protein, relative luciferase activities, and melanin in the control and experimental groups were analyzed by ANOVA and Fisher’s protected least significant difference test (SPSS 11.5 software). Data are reported as means ± sd. Values of *P* < 0.05 were significant.

## RESULTS

### Localization of microRNA-5110 in alpaca melanocytes

**Figure 1:**
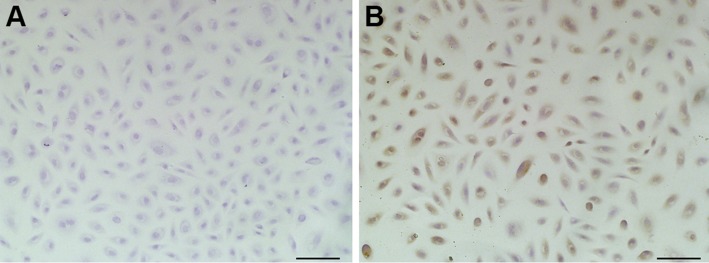
Localization of microRNA-5110 in alpaca melanocytes by *in situ* hybridization analysis. Melanocytes hybridized with NC (*A*) or digoxigenin-labeled microRNA-5110 (*B*) probe. Scale bars, 100 µm.

The localization of microRNA-5110 in alpaca melanocytes was studied *in vitro* with a specific *in situ* hybridization probe and a scrambled NC probe. A high level of specific microRNA-5110 probe hybridization was detected in the cytoplasm of these cells, whereas the NC scrambled probe produced no hybridization signal (**Fig. 1**). Compared with the alpaca keratinocytes, the expression of microRNA-5110 was higher in alpaca melanocytes (Supplemental Fig.1).

### Expression of microRNA-5110 in alpaca skin and melanocytes

**Figure 2:**
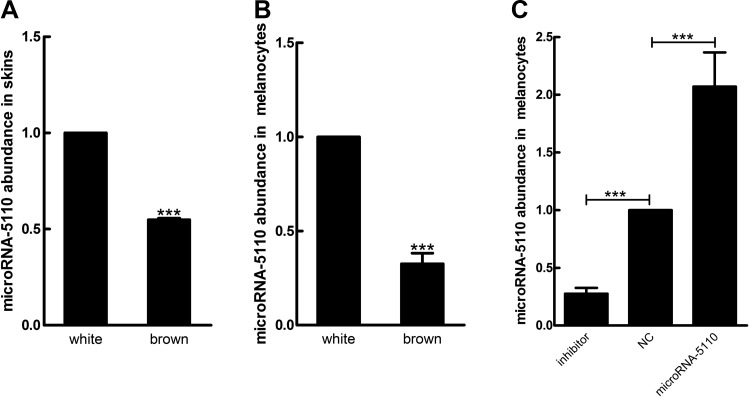
RT-qPCR analysis of microRNA-5110 in alpaca skin and melanocytes. *A*, *B*) The relative expression of microRNA-5110 in skin with white and brown coat color (*A*) and melanocytes (*B*). *C*) The relative expression of microRNA-5110 in alpaca melanocytes transfected with the microRNA-5110 expression and inhibitor plasmids. Data are expressed as means ± sd (*n* = 3). ***P < 0.001.

Compared with that in alpaca skin with white coat color, the expression level of microRNA-5110 was significantly lower in alpaca skin with brown coat color (**Fig. 2*A***). We tested the expression level of microRNA-5110 in melanocytes derived from skin with white and brown coat color and found that it was significantly lower in melanocytes of alpaca skin with brown coat color than in those of alpaca skin with white coat color (Fig. 2*B*). The transfection efficiency of synthetic microRNA oligonucleotides was tested by transfecting them into cultured melanocytes, and evaluating the expression of microRNA-5110 using quantitative reverse transcription-PCR. The expression level of microRNA-5110 was significantly increased in melanocytes transfected with microRNA-5110, as compared to those transfected with the control microRNA, and the expression level was significantly decreased in melanocytes transfected with the inhibitor (Fig. 2*C*). This result demonstrated that the microRNA-5110 plasmids used in this study were effective and could be used for subsequent experiments.

### MicroRNA-5110 targeting MLPH and WNT1

**Figure 3:**
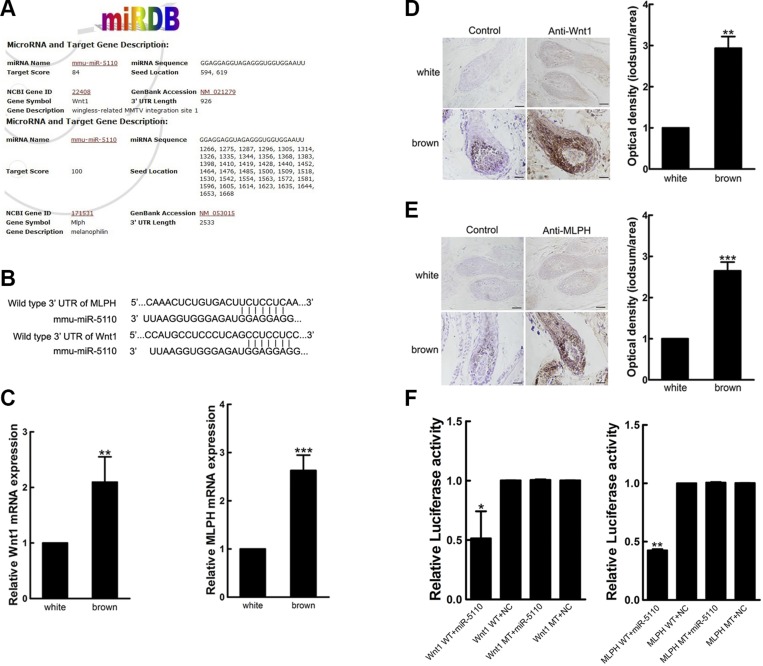
WNT1 and MLPH are microRNA-5110 targets. *A*) The microRNA-5110 binding sites in the 3ʹ-UTRs of WNT1 and MLPH, as predicted by miRBase software. *B*) The sequences of the wild-type 3ʹ-UTRs of WNT1 and MLPH and of microRNA-5110. *C*) The relative expression of WNT1 and MLPH in alpaca skin with white and brown coat color. *D*, *E*) Analysis of WNT1 (*D*) and MLPH (*E*) protein expression in alpaca skin with white and brown coat color. *F*) Dual luciferase gene assay data, expressed as the mean relative luciferase activities ± sd (*n* = 3). *P < 0.05, **P < 0.01, ***P < 0.001.

Bioinformatics analysis with miRBase software revealed that *MLPH* and *WNT1* mRNA were potential targets of microRNA-5110 (**Fig. 3*A, B***). RT-qPCR and immunohistochemical analyses showed that the expression of MLPH and WNT1 was higher in skin with brown coat color than in skin with white coat color (Fig. 3*C–E*). To confirm that microRNA-5110 bound to the 3ʹ-UTR of *MLPH* and *WNT1*, we constructed plasmids containing wild-type or mutant 3ʹ-UTRs of *MLPH* and *WNT1*. Cotransfection of these plasmids into HEK293T cells showed that microRNA-5110 significantly decreased the luciferase activity associated with the wild-type 3ʹ-UTR of *MLPH* or *WNT1*, as compared to the activity associated with the mutant 3ʹ-UTRs (Fig. 3*E, F*). These data indicate that microRNA-5110 can bind and regulate the levels of MLPH and WNT1 protein in a sequence-specific manner by interacting with its predicted 3ʹ-UTR binding sites.

### Effect of microRNA-5110 overexpression and knockdown on mRNA and protein levels of MLPH and WNT1

**Figure 4:**
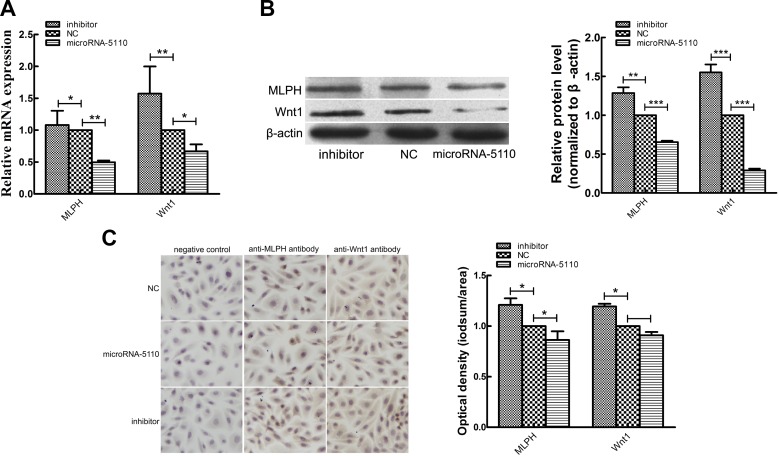
The effects of microRNA-5110 on WNT1 and MLPH mRNA and protein levels. *A*) Real-time qPCR analysis of MLPH and WNT1 in melanocytes transfected with the microRNA-5110 expression and inhibitor plasmids. Data are expressed as means ± sd (*n* = 3). Analysis of MLPH and WNT1 protein expression in melanocytes transfected with the microRNA-5110 expression and inhibitor plasmids by Western blot detection (*B*) and immunocytochemistry (*C*), respectively. *P < 0.05, **P < 0.01, ***P < 0.001.

The overexpression and knockdown of microRNA-5110 in alpaca melanocytes reduced and increased, respectively, the levels of mRNAs encoding *MLPH* and *WNT1* (**Fig. 4*A***). Western blot analysis and immunohistochemical analyses also showed that the levels of MLPH and WNT1 proteins were reduced and increased in melanocytes by overexpression and knockdown of microRNA-5110, respectively (Fig. 4*B, C*). These data indicate that MLPH and WNT1 expression are regulated by microRNA-5110.

### Effect of microRNA-5110 overexpression and knockdown on the expression of melanogenic genes

**Figure 5:**
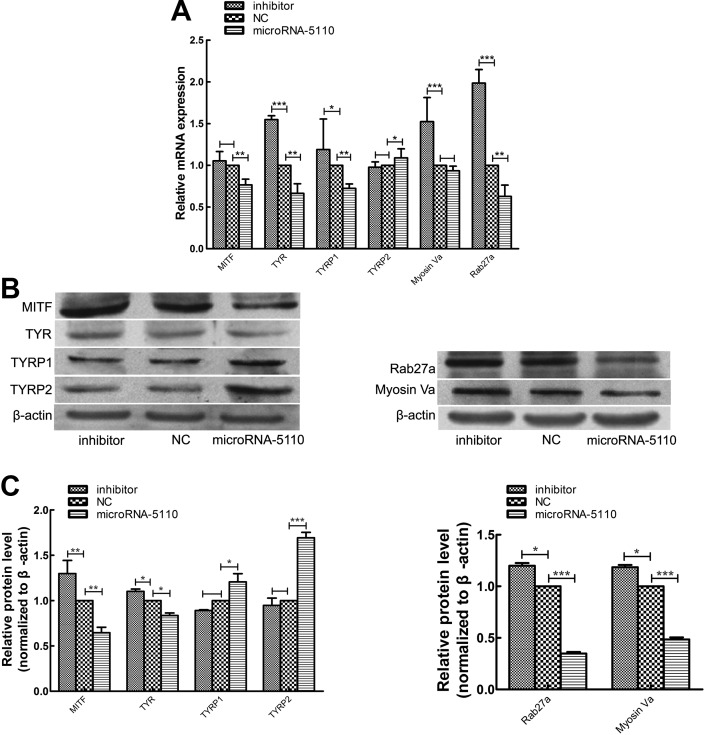
The effects of microRNA-5110 on pigmentation in melanocytes. *A*) The mRNA expression of coat color genes in melanocytes. *B*) Immunoblotting of β-actin, MITF, TYR, TYRP1, TYRP2 RAB27a and MYO5a in the indicated melanocytes. *C*) The protein expression of MITF, TYR, TYRP1, TYRP2, RAB27a and MYO5a. Data are expressed as means ± sd. *P < 0.05, **P < 0.01, ***P < 0.001.

To explore the effects of microRNA-5110 on melanogenesis in alpaca melanocytes, we investigated the expression levels of genes involved in this process. Overexpression and knockdown of microRNA-5110 in melanocytes resulted in reduced and increased levels of mRNAs encoding *RAB-27a*, *MYO-5a*, *MITF*, *TYR*, and *TYRP-1*, respectively, whereas the level of *TYRP2* mRNA was significantly increased and decreased, respectively (**Fig. 5*A***). Western blot analysis showed that the expression of RAB27a, MYO5a, MITF, and TYR proteins was significantly reduced and increased, whereas the expression of TYRP1 and 2 proteins were significantly increased and decreased in melanocytes with microRNA-5110 overexpression and knockdown, respectively (Fig. 5*B, C*).

### Effect of microRNA-5110 overexpression and knockdown on melanin production

**Figure 6:**
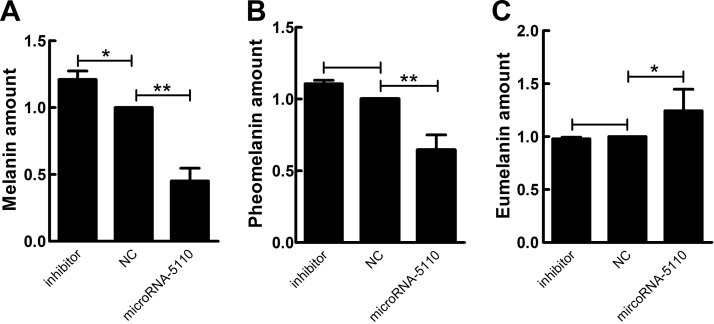
Effect of microRNA-5110 on melanin production in alpaca melanocytes. The levels of total alkali melanin (*A*), pheomelanin (*B*), and eumelanin (*C*) in melanocytes after overexpression and knockdown of microRNA-5110. Data are expressed as the means ± sd (*n* = 3). *P < 0.05, **P < 0.01.

To investigate whether microRNA-5110 affects melanin production, total alkali melanin, eumelanin, and pheomelanin were measured in alpaca melanocytes after overexpression and knockdown of microRNA-5110. Overexpression and knockdown of microRNA-5110 reduced by 45% and increased by 20% the total alkali melanin level, respectively, and reduced by ∼64% and increased by ∼10% the pheomelanin level, respectively (**Fig. 6*A, B***). However, the eumelanin level increased by ∼24% in cells transfected with microRNA-5110 (Fig. 6*C*). To investigate whether the effect of microRNA-5110 on melanin production is species specific, we also measured the changes in melanin production in mouse melanocytes after overexpression and knockdown of microRNA-5110. The results showed that the total alkali melanin level was reduced by 29%, the pheomelanin level was reduced by 34%, and the eumelanin level was increased by 35% in mouse melanocytes overexpressing microRNA-5110. In contrast, the total alkali melanin level was increased by 13%, the pheomelanin level was increased by 14%, and the eumelanin level was reduced by 28% in melanocytes after knockdown of microRNA-5110. These findings are similar to the changes in melanin production in alpaca melanocytes, which suggests that the effects of microRNA-5110 on melanin production are not specific to species (Supplemental Fig. 2).

## DISCUSSION

Melanocytes are neural crest–derived cells that synthesize and store melanin within unique lysosome-related organelles, termed melanosomes (10). Melanin is responsible for skin and hair color in mammalian species. The phenotype of hair color depends on the levels and ratio of 2 types of melanin (black–brown eumelanin and yellow–red–brown pheomelanin) produced by resident skin melanocytes (11, 12). Many precise mechanisms play important roles in the determination of skin or hair color, including microRNAs. To date, the expression and function of microRNAs has been investigated in the skin of several mammalian species, including mouse (13, 14), goat, sheep (15), and alpaca (16, 17). Some microRNAs are known to affect the hair color phenotype. For example, overexpression of microRNA-137 in mice changes their skin color from black to brown (18). Other microRNAs known to be involved in the pigmentation process include microRNA-340 and -145 (19, 20).

Deep sequencing of microRNAs in alpaca skin identified 15 and 18 potentially novel microRNAs from white and brown skin, respectively, and some microRNAs, such as microRNA-5110, showed significantly different expression levels in white *vs.* brown skin (6). The present study has provided information on the higher expression of microRNA-5110 in alpaca melanocytes than that in keratinocytes, which suggests that microRNA-5110 is related to the melanin production and pigmentation. Consistent with the miRBase output, microRNA-5110 overexpression inhibited the luciferase activity from plasmids encoding the MLPH and WNT1 wild-type 3ʹ-UTRs, but not from those encoding mutated 3ʹ-UTRs. Furthermore, overexpression of microRNA-5110 downregulated the expression of MLPH and WNT1 mRNA and protein in melanocytes. These findings indicate that *MLPH* and *WNT1* are target genes of microRNA-5110 in melanocytes.

MLPH belongs to the synaptotagmin-like protein family and more precisely to a branch of sequences that lack C2 domains (also designated as SLAC2) (21–23). MLPH is a critical component of the melanosome transport machinery (24) and has been characterized as a linker protein in a complex formed by MYO5a and RAB27a that plays an important role in the capture and short-range actin-based delivery of melanosomes to the melanocyte periphery (21, 25–27). In the present study, the mRNA and protein expression levels of *MYO5A* and *RAB27A* were reduced in melanocytes with lower MLPH levels after overexpression of microRNA-5110. MLPH could be involved in later steps of melanosome transport, such as vesicle targeting and vesicle fusion: binding of MLPH to activated RAB27a could inhibit melanosome fusion with target membranes, which would prevent release of pigment granules to neighboring keratinocytes (24). Thus, microRNA-5110 would affect melanosome transport through the regulation of MLPH.

In the current study, we found that overexpression of microRNA-5110 in alpaca melanocytes downregulated the expression of MITF *via* WNT1, especially at the protein level. WNT signaling pathways, which have high evolutionary conservation from *Drosophila* to humans, are involved in a variety of cellular functions ranging from embryonic development to adult homeostasis and tumor progression (28). *WNT* proteins comprise a large family of highly conserved growth factors that are responsible for important developmental and homeostatic processes throughout the animal kingdom (29). To date, 3 WNT signaling pathways have been characterized: the canonical WNT/β-catenin pathway regulates the transcription of a broad spectrum of target genes, the noncanonical WNT/calcium pathway controls intracellular calcium levels, and the noncanonical planar cell polarity pathway regulates cytoskeleton rearrangement (30, 31). WNT1 is involved in the canonical WNT/β-catenin pathway, which results in reduced cytoplasmic degradation of β-catenin, then interacts with a protein complex in the nucleus to regulate gene transcription (32). β-Catenin associates with members of the LEF/TCF family of transcription factors to control the level of *MITF* gene transcription. MITF plays a fundamental role in melanocyte development and maintenance and seems to be crucial for the survival of malignant melanocytes (33). Furthermore, MITF can regulate melanocyte cellular differentiation and the transcription of melanogenic enzymes, including TYR, TYRP1, and TYRP2 (1, 34). However, a previous report indicated that MITF may not be a principal regulator of the TYRP2 gene (35). TYR is absolutely required for both eumelanin and pheomelanin synthesis, whereas TYRP1 and 2 seem to be more crucial for eumelanin synthesis (36). The present result of the downregulation of TYR and upregulation of TYRP2 *via* microRNA-5110 made the contribution to the decreased total melanin production and pheomelanin production and increased eumelanin production. The mRNA expression of TYRP1 was decreased, but its protein expression was increased. In fact, TYRP1 expression is an intriguing feature: it has been reported that the transcription of TYRP1 is frequently and selectively attenuated and completely extinguished in melanoma cell lines (37). Further research is needed to identify the mechanism. To verify the effect of microRNA-5110 on melanin production, we conducted the melanin content assay in mouse melanocytes transfected with microRNA-5110 and got the same result as that in alpaca melanocytes.

## CONCLUSIONS

This work demonstrates that microRNA-5110 regulated RAB27a/MYO5a and WNT/β-catenin signaling pathways by cotargeting *MLPH* and *WNT1,* which resulted in up-regulated eumelanin production through TYRP2 and down-regulated pheomelanin production through TYR in alpaca melanocytes.

## Supplementary Material

This article includes supplemental data. Please visit *http://www.fasebj.org* to obtain this information.

## Abbreviations

MITF: microphthalmia-associated transcription factor
MLPH: melanophilin
qPCR: quantitative PCR
RAB27a: rat sarcoma (RAS)–associated binding 27a
MYO5a: myosin 5a
TYR: tyrosinase
TYRP: tyrosinase-related protein
WNT1: WNT family member 1
WT: wild type
