# Supplementary figures and images for: MicroRNA-5110 regulates pigmentation by cotargeting melanophilin and WNT family member 1
Source: FASEB J. 2018 May 7;32(10):5405–12. doi: 10.1096/fj.201800040R (PMC6133708; doi:10.1096/fj.201800040R)

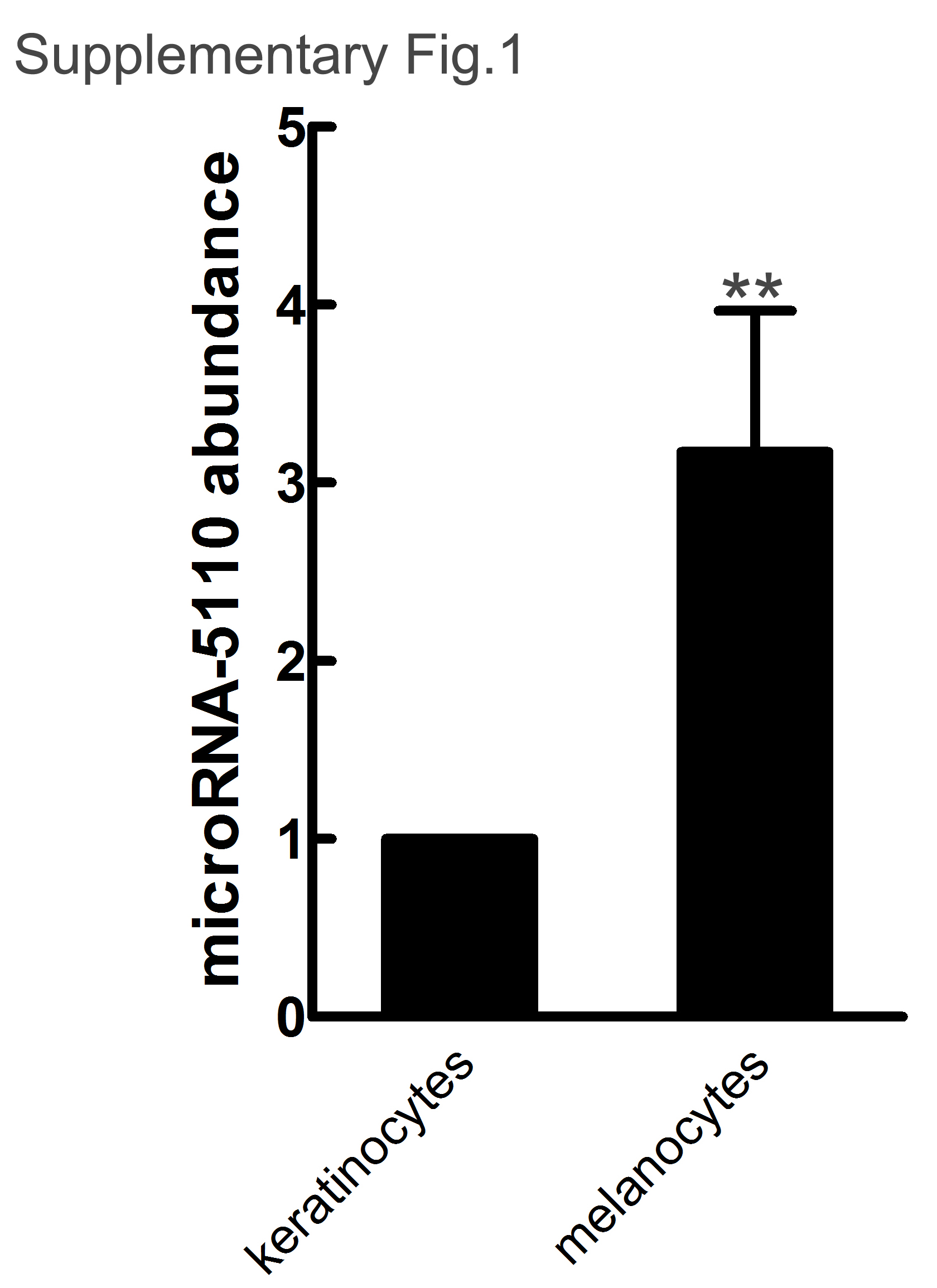

Supplement: Supplementary file 1 [file fj.201800040R.sf1.jpg]

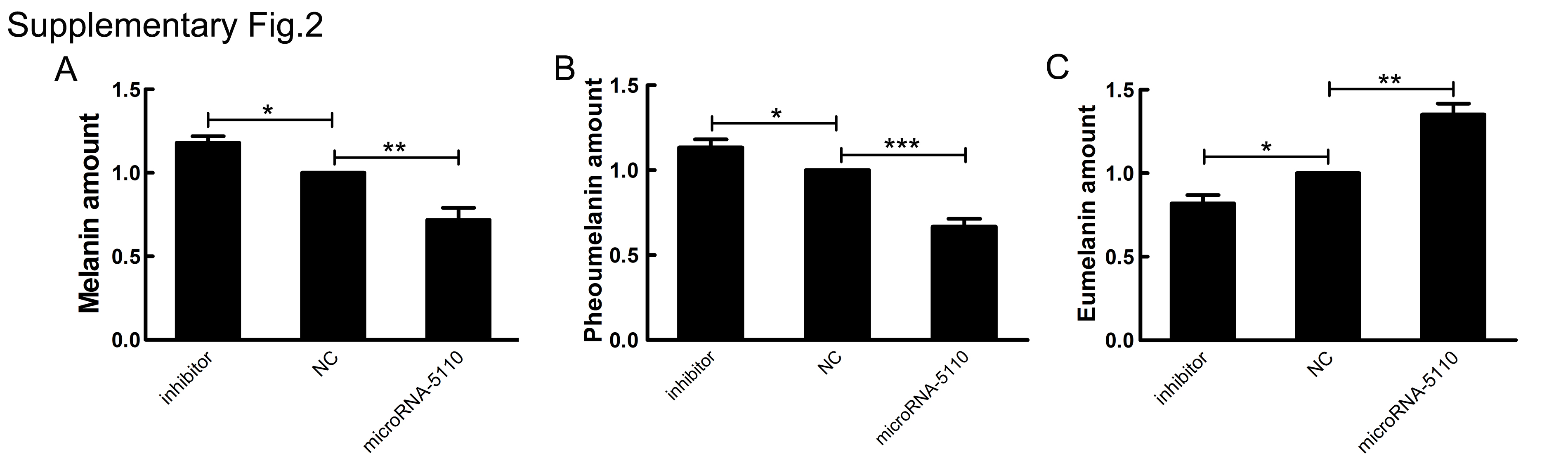

Supplement: Supplementary file 2 [file fj.201800040R.sf2.jpg]
