# Supplementary material for: MicroRNA-5110 regulates pigmentation by cotargeting melanophilin and WNT family member 1
Source: FASEB J. 2018 May 7;32(10):5405–12. doi: 10.1096/fj.201800040R (PMC6133708; doi:10.1096/fj.201800040R)
Supplement: Supplementary file 3 [file fj.201800040R.sd1.docx]

**Supplementary Figure 1. RT-qPCR analysis of microRNA-5110 in melanocytes and keratinocytes of alpaca.** Data are expressed as means ± standard deviations (n = 3), *P < 0.05.

**Supplementary Figure 2. Effect of microRNA-5110 on melanin production in mouse melanocytes.** The levels of (A) total alkali melanin, (B) pheomelanin, and (C) eumelanin in melanocytes after overexpression and knockdown of microRNA-5110. Data are expressed as the means ± standard deviations (n = 3); *P < 0.05; **P < 0.01. NC, negative control.
